# Determination of the Spatial Anisotropy of the Surface MicroStructures of Different Implant Materials: An Atomic Force Microscopy Study

**DOI:** 10.3390/ma14174803

**Published:** 2021-08-24

**Authors:** Alessandro Gambardella, Gregorio Marchiori, Melania Maglio, Alessandro Russo, Chiara Rossi, Andrea Visani, Milena Fini

**Affiliations:** 1 Struttura Complessa Scienze e Tecnologie Chirurgiche, IRCCS Istituto Ortopedico Rizzoli, 40136 Bologna, Italy; gregorio.marchiori@ior.it (G.M.); melania.maglio@ior.it (M.M.); andrea.visani@ior.it (A.V.); milena.fini@ior.it (M.F.); 2 Clinica Ortopedica e Traumatologica 2, IRCCS Istituto Ortopedico Rizzoli, 40136 Bologna, Italy; alessandro.russo@ior.it (A.R.); chiara.rossi@ior.it (C.R.)

**Keywords:** biomaterials, prosthetics, implants, anisotropy, titanium alloy, fractal analysis, roughness, atomic force microscopy

## Abstract

Many biomaterials’ surfaces exhibit directional properties, i.e., possess spatial anisotropy on a range of spatial scales spanning from the domain of the naked eye to the sub-micrometer level. Spatial anisotropy of surface can influence the mechanical, physicochemical, and morphological characteristics of the biomaterial, thus affecting its functional behavior in relation, for example, to the host tissue response in regenerative processes, or to the efficacy of spatially organized surface patterns in avoiding bacterial attachment. Despite the importance of the availability of quantitative data, a comprehensive characterization of anisotropic topographies is generally a hard task due to the proliferation of parameters and inherent formal complications. This fact has led so far to excessive simplification that has often prevented researchers from having comparable results. In an attempt to overcome these issues, in this work a systematic and multiscale approach to spatial anisotropy is adopted, based on the determination of only two statistical parameters of surface, namely the texture aspect ratio *S_tr_* and the roughness exponent *H*, extracted from atomic force microscopy images of the surface. The validity on this approach is tested on four commercially available implant materials, namely titanium alloy, polyethylene, polyetheretherketone and polyurethane, characterized by textured surfaces obtained after different machining. It is found that the “two parameters” approach is effective in describing the anisotropy changes on surfaces with complex morphology, providing a simple quantitative route for characterization and design of natural and artificial textured surfaces at spatial scales relevant to a wide range of bio-oriented applications.

## 1. Introduction

The surface of many implant materials is often marked with lays originating from various machining and finishing processes. Such lays can appear oriented along one preferential direction or exhibit patterns sometimes very difficult to describe visually. The presence of directional properties is commonly referred to as spatial anisotropy or roughness anisotropy of surface; of course, it is the opposite of isotropic textures of surfaces without a really dominating direction, such as some grit-blasted or etched ones [1,2,3,4].

In regenerative medicine, anisotropic biomaterials’ surfaces may be preferred for developing tissue-engineering constructs as they present morphology and function more closely resembling the native tissue [5]. In particular, their role has been highlighted in relation to architectural reconstruction and scaffold design of blood vessels [6] and skeletal muscle tissue [5,6,7]. Typically, geometries of biomaterials for tissue anisotropy reconstruction can be formed as orientated fibers produced via electrospinning and flow shear, or micropatterning of substrates obtained through methods such as lithography, soft lithography, direct laser writing and abrasion wear [7]. 

Strategies of surface topography modification via creation of micro patterns have attracted attention in relation to osseointegration of implants, in order to mimic the surrounding biological environment as well as reduce the inflammation/infection that may occur [8,9,10,11,12,13]. 

Not least, an important field of study investigates the role of engineered topographies in the phenomenology of the so-called "race for surface" in relation to decreased bacterial adhesion in implant infections [14,15,16,17,18,19]. 

Additionally, coating of implants for purposes of improved bioactivity, osseointegration or antibacterial properties may alter the original microstructure of textured surfaces and then affect directional properties of the surface, especially at the micrometer scales [20,21,22]. 

In dealing with implants’ surfaces, mechanical, topographic, and physicochemical properties such as wettability are frequently related, and thus changing one means changing the others as well [23,24,25,26,27]. In addition, spatial anisotropy may affect different scales in different ways, and then affect the performance of an implant material at different scales in different ways [4]. Despite these issues, a significant drawback of studies involving spatial anisotropy of implants’ surfaces is that they did not provide sufficiently detailed quantitative topographical data. For example, textured surfaces have been extensively characterized solely by height, or amplitude parameters such as the average or root mean square roughness (*R_a_* or *R_s_*, respectively); however, surfaces with different topographies may have the same *R_a_* or *R_s_*, and thus these quantities alone cannot be used effectively in dealing with anisotropic surfaces. In this respect, the need for spatial parameters in addition to height parameters was highlighted, in particular, in studies that related the bacterial response to spatially organized micro topographies [16,18,19]. In these works, however, it is implicit that the variety of manufacturing methods and forms of texture the various processes generate lead to a proliferation of parameters that requires simplification in the experimental and analytical methods of surface investigation. 

This paper is based on an overview of the literature concerning extraction of roughness and spatial parameters through methods originally developed in the engineering field for quantitatively characterizing machined/finished surfaces, mainly for tribological applications [1,2,3,4,28]. Generally speaking, the extraction of parameters from a given surface implies that topographic maps may be acquired by ellipsometry, profilometry or scanning probe techniques, and then analyzed by statistical methods over a given range of spatial scales. For implant materials, such range falls typically around the cell dimension (tens of micrometers or smaller), hence in the domain of atomic force microscopy (AFM). Among other techniques, AFM offers the advantages of having high spatial resolution and of being applicable, within certain limits, to potentially every kind of material, which is very attractive for many practical purposes. 

A first, classical approach to technical surfaces characterizes quantitatively the evolution of the roughness towards small scales via the so-called Hurst exponent *H* [29,30,31,32,33,34,35]. On surfaces with no directional properties, it has a unique value *H_iso_* that can be determined through various methods on a given interval of scan lengths. In contexts that completely disregarded any directionality of surface, *H_iso_* has become popular to monitor and compare the evolution of the morphology of many technical surfaces after different manufacturing processes [31,32,33]. On the other hand, for surfaces possessing strong directional properties the roughness decay with the length scale depends on the directions [1,2,33,36,37], resulting in a hybrid between amplitude and spatial parameter which can be determined from the two-dimensional power spectral density function of the surface heights (*PSD*) [1,28,29,30]. In other words, the *PSD* is sensitive to anisotropy and describes, on a given portion of surface, how *R_s_* decays along a desired direction or angle *α*. The so determined directional *H* will be referred to in the following as to *H_α_*. 

As a spatial parameter, the texture aspect ratio *S_tr_* of the surface can be easily extracted from the two-dimensional autocorrelation function of a surface (*ACF*) [16,28,29,30]. Such a function is highly sensitive to spatial anisotropy and can be used to determine the dominant direction of anisotropy, while *S_tr_* measures its strength. Note that *PSD* and *ACF* represent a Fourier transform pair and can be applied independently to the same topographic image for extracting *H_α_* and *S_tr_*, respectively. A set of images taken at different scan sizes *L* will show, on average, the scale evolution of these parameters. 

The objective of the present work is to develop a systematic approach to multiscale anisotropy, and to test it on commercially available implant materials. Thus, in the following, *H_iso_*, *S_tr_* and *H_α_* will be extracted from AFM topographies of four implant materials with textured surfaces. In particular, two samples, namely titanium alloy (Ti-6Al-4V) and ultra-high molecular weight polyethylene (UHMWPE) exhibited a strong directional morphology, while polyetheretherketone (PEEK) and polyurethane (PU) samples showed a less evident lay. In the light of the results obtained here, and within some limitations detailed in the following, we found that for surfaces possessing a single lay our approach accounts well for the micro topographies observed, and highlights that the anisotropy changes towards small scales are determined by the interplay between the inheritance of the machining/finishing processes and the spatial distribution of features specific to the sample. 

## 2. Materials and Methods

### 2.1. Materials Tested and Their Surfaces

**Figure 1:**
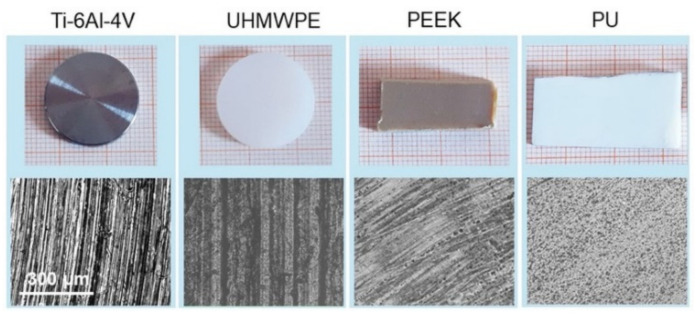
The four materials used in this work (**top**) and (**bottom**) corresponding 20× optical images of their surfaces. The scale bar on the left is the same for all the images.

Pictures of the samples selected for this study are shown in Figure 1 along with their corresponding 20× optical images. Ti-6Al-4V is of very common use in orthopedics, given its excellent mechanical properties, corrosion resistance and good biocompatibility [38]. In the same field, UHMWPE is one of the most widely used materials for the bearing surface of prostheses in hip replacement, sometimes with adequate functionalization to overcome limitations related essentially to wear debris [39]. PEEK has emerged over the years as a promising material for prostheses, partially due to its ability to be processed using additive manufacturing techniques [40,41]. PU is used for general purpose catheters and tubes; in general, surface modifications (e.g., with antibacterial, hydroxyapatite, or extracellular matrix components) have often been necessary to improve its bioactivity [20,42]. In this work, Ti-6Al-4V and UHMWPE were face-turned disks (20 mm diameter, Citieffe S.r.l., Calderara di Reno, Bologna, Italy), marked with a clearly visible circular lay. This leads to a morphology constituted essentially by regularly spaced grooves. PEEK and PU were medical-graded sheets (6 mm height, Direct Plastics Ltd., Sheffield, UK) with semi-machined surface; here the texture is barely recognizable by the naked eye, although the optical images allow us to visualize an effective degree of surface orientation. Note that, for most applications, further processing may be needed for the surfaces above; indeed, they were studied in the present form uniquely for the purpose of having different biomaterials with different degrees of spatial anisotropy to test the range of validity of the adopted approach. 

### 2.2. Roughness Meter and AFM Operation

A roughness meter (PCE-RT1200 model, PCE Instruments S.r.l., Capannori, Lucca, Italy) was used to evaluate one-dimensional root-mean-square roughness *R_s_*(*l*) at *l* = 2.5, 0.8 and 0.25 mm. Surface topographies with lateral size ranging from *L* = 100 to 2 µm were acquired by a standalone atomic force microscope (NT-MDT Co., Moscow, Russia) equipped with Si cantilevers (tip curvature radius ~ 10 nm, resonant frequency ~ 240 kHz) and operating in tapping mode in air and at room temperature. All images were recorded at N = 512 × 512 points and unfiltered, except for a 2nd order levelling. *R_s_(L)* values, *ACF* and *PSD* functions and related parameters *H_iso_*, *S_tr_* and *H_α_* were extracted from topographies via Gwyddion software (version 2.58, Czech Metrology Institute, Brno, Czech Republic) over 7 non-overlapped images for each scan size. All samples were measured after ultrasonic cleaning in isopropyl alcohol for 5 min and subsequent drying by nitrogen flow.

### 2.3. Extraction of Surface Parameters from AFM Images

#### 2.3.1. Isotropic Hurst Exponent H_iso_

Let us consider a square portion of surface of size *L* and be *R_s_*(*L*) the corresponding roughness value. Most technical surfaces satisfy the so-called relation of self-affine scaling:

(1) $$R_{s}\left(L\right)\propto L^{H_{iso}},$$

where the subscript *iso* refers to the isotropic character of Equation (1). Typically, the (1) exhibits an onset of abscissa *ξ* (correlation length). The regime of self-affine scaling is valid for *L* << *ξ* while, for *L* >> *ξ*, *R_s_*(*L*) tends to reach saturation. According to the so-called roughness method [31], *H_iso_* can be evaluated from the linear fitting of the Log-Log plot of the (1) at *L* << *ξ*. Note that 0 < *H_iso_* < 1; it essentially measures how much the surface ruggedness changes towards small length scales, so that *H_iso_* closer to 0 means very jagged surface while closer to 1 means a smoother surface [3,31,32,43]. 

#### 2.3.2. Texture Aspect Ratio S_tr_

**Figure 2:**
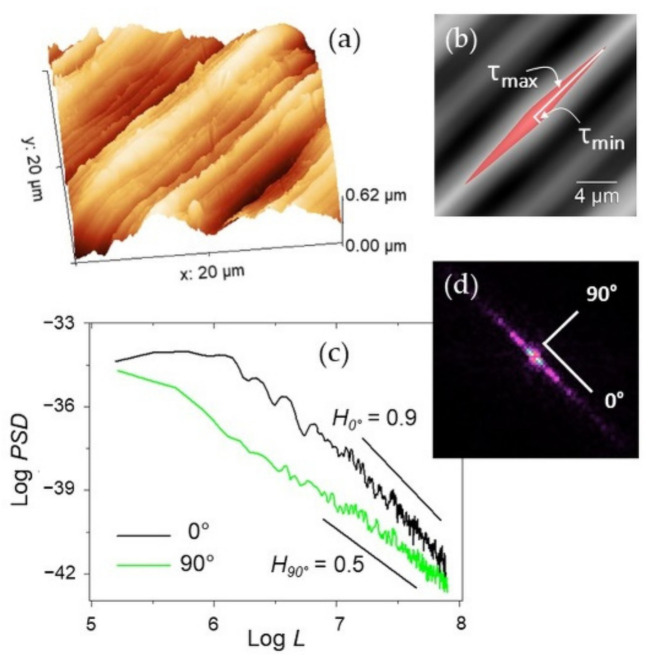
(**a**) Representative 20 × 20 µm^2^ AFM image of Ti-6Al-4V and (**b**) its autocorrelation image with indication of the central lobe (red area) and of the corresponding minimum and maximum lag *τ_min_* and *τ_max_*. (**c**) A Log-Log plot of the *PSD* functions calculated over the same image along the lay (0° direction, black curve) and across it (90°, green curve) with indication of the different slopes obtained along the corresponding directions. (**d**) FFT of the image in (**a**).

The surface topography recorded in the form of discrete height samples *z*(*i*, *j*) of an AFM image allows to compute the *ACF* function as [3,36,44,45]:

(2) $$ACF\left(\tau_{x},\tau_{y}\right)=\overset{\infty }{\underset{-\infty }{\iint }}z_{1}z_{2}w\left(z_{1},z_{2},\tau_{x},\tau_{y}\right)dz_{1}dz_{2}$$

where *z*_1_ and *z*_2_ are the values of heights at points (*x*_1_, *y*_1_), (*x*_2_, *y*_2_); furthermore, *τ_x_* = *x*_1_ − *x*_2_ and *τ_y_* = *y*_1_ − *y*_2_ are the distances between these points, or spatial lag along scan axes *x* and *y*. The function *w*(*z*_1_, *z*_2_, *τ_x_*, *τ_y_*) denotes a two-dimensional probability density corresponding to points (*x*_1_, *y*_1_), (*x*_2_, *y*_2_) [3,36,44,45]. The *ACF* is sensitive to non-randomness in topographic data, and thus can be used on morphologies with repeating features to describe the horizontal distribution of roughness. It can be easily extracted via software from AFM images such as in Figure 2a. The *ACF* is a symmetric, real function, with the maximum value at the origin (zero lag). It follows the periodicity of the surface and asymptotically decays toward zero with increasing spatial lag. The minimum (*τ_min_*) and maximum (*τ_max_*) radii are sought on the image of the central lobe generated by thresholding the central autocorrelation peak (see Figure 2b). If the surface presents the same characteristics in every direction, the central lobe will be approximately circular, and *τ_min_* ~ *τ_max_*. If the surface presents a strong privileged orientation, as in the example shown, the central lobe will be very stretched out and *τ_max_* >> *τ_min_*. The texture aspect ratio *S_tr_* is then defined as [16,36]:

(3) $$0\leq S_{tr}=\frac{\tau_{min}}{\tau_{max}}\leq 1$$

For *S_tr_* > 0.5 the surface is said to be isotropic, while for *S_tr_* < 0.3 the surface is said to be strongly anisotropic. 0.3 < *S_tr_* < 0.5 denotes a surface with intermediate characteristics [20,30]. Note that *S_tr_* is determined by the decay of (2) over a fraction of the total image area; the cut-off threshold is usually set to values from 0.1 to 0.5, so that points below the threshold are considered essentially uncorrelated. In this work, the threshold was chosen higher or lower than 0.2 value to ensure that the central lobe is well defined and does not touch the edges of the image [44].

#### 2.3.3. Directional Roughness Exponent 

On anisotropic surfaces, where the roughness decay depends on the directions, one has to consider the Fourier transform of the *ACF*, or two-dimensional power spectral density of surface heights:

(4) $$PSD\left(k_{x},k_{y}\right)=\overset{\infty }{\underset{-\infty }{\iint }}ACF\left(\tau_{x},\tau_{y}\right)e^{-i\left(k_{x}\tau_{x}+k_{y}\tau_{y}\right)}d\tau_{x}d\tau_{y}$$

where *k* is a vector in the reciprocal space of components (*k_x_*, *k_y_*). On a self-affine, randomly rough surface Equation (4) takes a particularly simple form at large *k* [45]:

(5) $$PSD\left(k\right)\propto k^{-2\left(H+1\right)}$$

where $k=\left|k\right|=\sqrt{k_{x}^{2}+k_{y}^{2}}$ is the vector in the reciprocal space that runs along the direction angle *α*. Note that for isotropic or weakly anisotropic surfaces *H* is essentially independent of *α*, while for a strongly anisotropic surface *H* will be higher in the direction parallel to the lay, or principal direction (*α* = 0°) while it will decrease slightly across the lay (*α* = 90°, measured counterclockwise) [1]. This information is illustrated by the different slopes of the curves in Figure 2c, which also evidences their characteristic self-affine decay at large *k*. For a given *α*, said *C_α_* the slope at large *k* of Equation (5), one has *H_α_* = −2(*C_α_* +1). Thus, *H_α_* can be extracted from the linear fit of the Log-Log plot of Equation (5) evaluated along the direction of the vector *k*. Note that the direction *α* = 0° runs along the maxima of the fast Fourier transform (FFT) in the reciprocal space, as highlighted in Figure 2d. Finally, it is underlined that the finite size of image algorithms that calculate *PSD* may provide size-dependent results [45,46]. As a consequence, *H_α_* extracted from single images cannot be, in general, used to compare images with different *L*. Thus, in this work the relative variations of *H_α_* (with respect to the value it assumes along the principal direction), i.e., the *H_α_*/*H_0°_* ratio to monitor its evolution with *L*, will be provided. 

## 3. Results

### 3.1. Surface Topographies and Roughness vs. Length Scale

**Figure 3:**
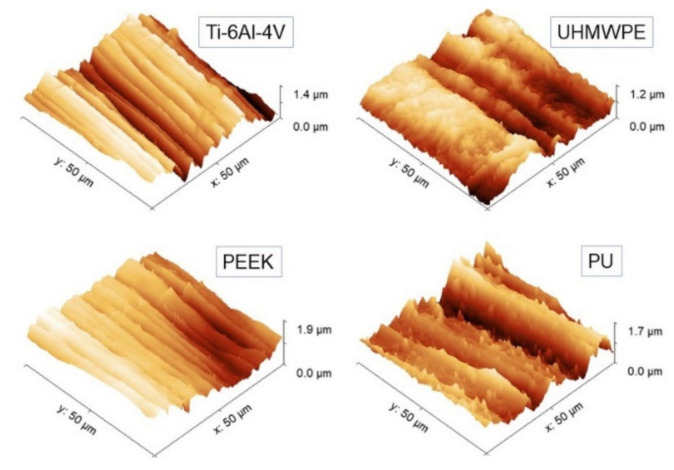
Representative 50 × 50 µm^2^ AFM topographies of the four surfaces investigated. All the images have been displayed at the same rotation angle for a guide to the eye.

**Figure 4:**
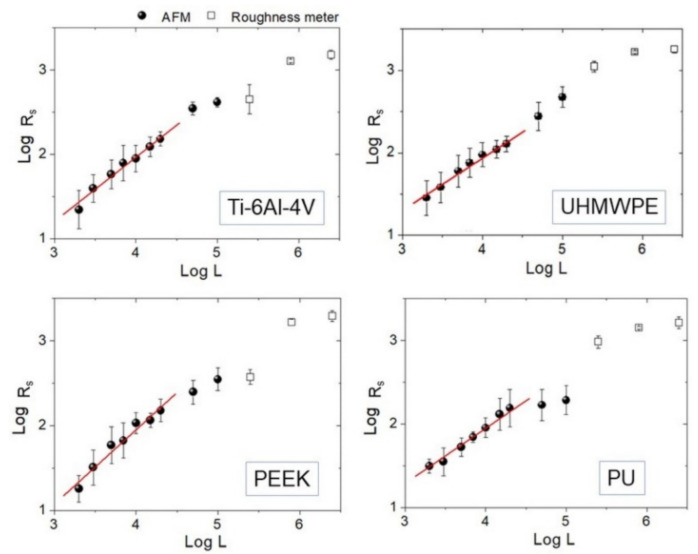
Log-Log plots of *R_s_* vs. *L* curve for each sample. Experimental points measured by AFM (black round dots) and roughness meter (square dots) are reported together. Linear fittings on AFM data below *L* = 20 µm are also highlighted.

Representative 50 × 50 µm^2^ AFM topographies of the four surfaces are shown in Figure 3. Beyond the visual impression given by the images, which essentially show the presence of a single lay, or a main direction of anisotropy, further details can be added by the analysis of the evolution of the *R_s_* vs. *L* curves according to paragraph 2.3.1 and reported in Figure 4. The four surfaces possess similar root-mean-square roughness (*R_s_* ~ 1.5–2.0 µm) at sub-millimeter distances, while different slopes characterize the Log-Log curves towards microscopic scales. Note that *ξ* falls around macroscopically large distances for the surfaces investigated here.

Then, *H_iso_* was estimated from the images using Equation (1), and the interval 2 ≤ *L* ≤ 20 µm consistently for all samples was chosen. The results of the linear fittings are displayed in decreasing order of value in Table 1. Note that the ratios of the standard deviation of the mean *H_iso_* to mean *H_iso_* range from about 4% to 8%, suggesting that, in the interval considered, the surfaces can be treated as self-affine to a good approximation. Note that—on average—PEEK has the smoothest profile of the set (highest *H_iso_*), followed by Ti-6Al-4V with which, according to Figure 3, it shares a very similar morphology. In contrast, PU and UHMWPE display clearly more jagged surfaces, as readily shown by the corresponding images. As shown in the following, the information gained from Table 1 will be helpful to better understand the scale evolution of the anisotropy-related parameters *S_tr_* and *H_α_*.

### 3.2. Texture Aspect Ratio vs. Length Scale

**Figure 5:**
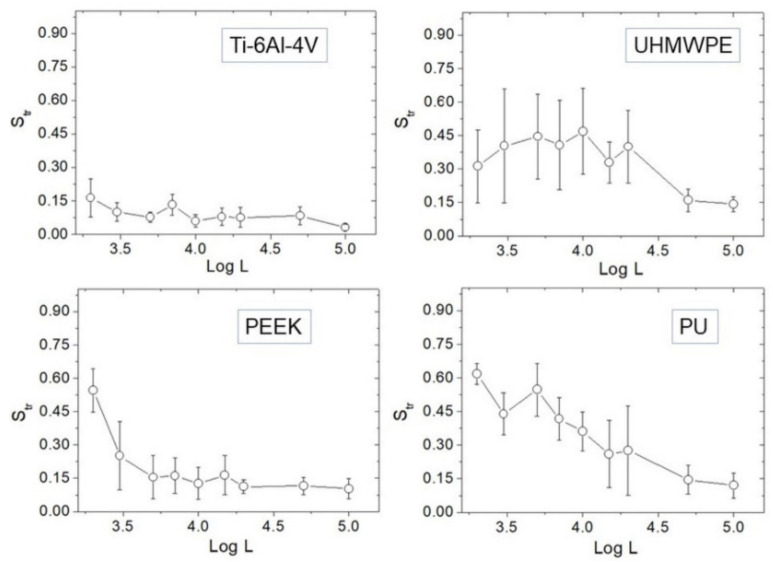
Plots of *S_tr_* against Log *L* for each sample.

The evolution of *S_tr_* vs. Log *L* is reported (Figure 5). At large scales, namely *L* = 50–100 µm, all surfaces are strongly anisotropic (*S_tr_*~0); however, distinct behavior characterizes the different surfaces towards small scales. Ti-6Al-4V tends to remain strongly anisotropic at all scales, besides a very slight tendency of *S_tr_* to increase. For UHMWPE, the anisotropy results mitigated below *L*~20 µm, although at smaller distances *S_tr_* did not increase further. 

**Figure 6:**
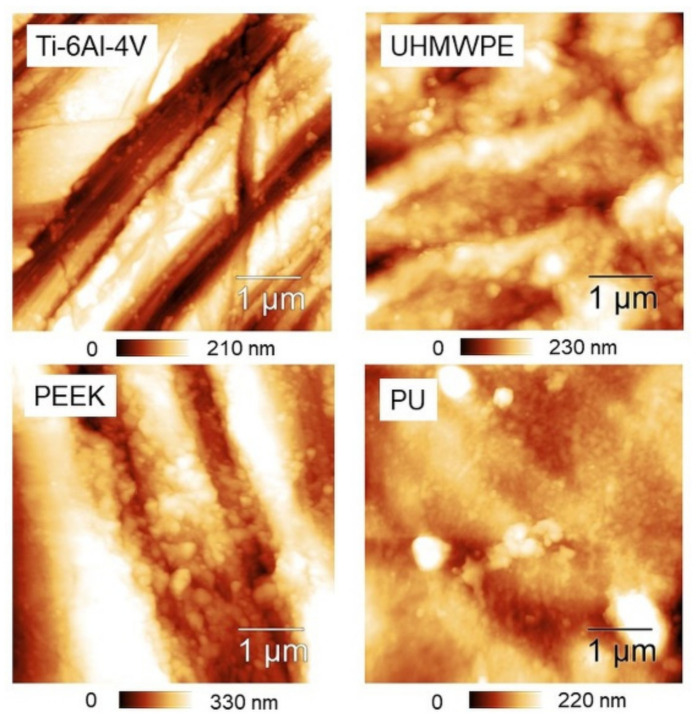
5 × 5 µm^2^ AFM topographies of the four surfaces.

In contrast, a clear tendency of *S_tr_* to increase towards small scales is found in PEEK and PU. If *S_tr_* ~ 0.5 is assumed as reference, one may say that—on average—PEEK is essentially isotropic below *L* = 2 µm, while PU is already below *L* = 5 µm. Representative 5 × 5 µm^2^ AFM images are also reported (Figure 6), showing that—especially for the first three samples—a directional character is still present at micrometer distances, although with remarkable differences from one surface to another. Before using Table 1 to go through these differences, as outlined in the Discussion section we test the effectiveness of using *H_α_* instead of *S_tr_* in dealing with anisotropy. 

### 3.3. Relative Variations of H as a Measure of Anisotropy

**Figure 7:**
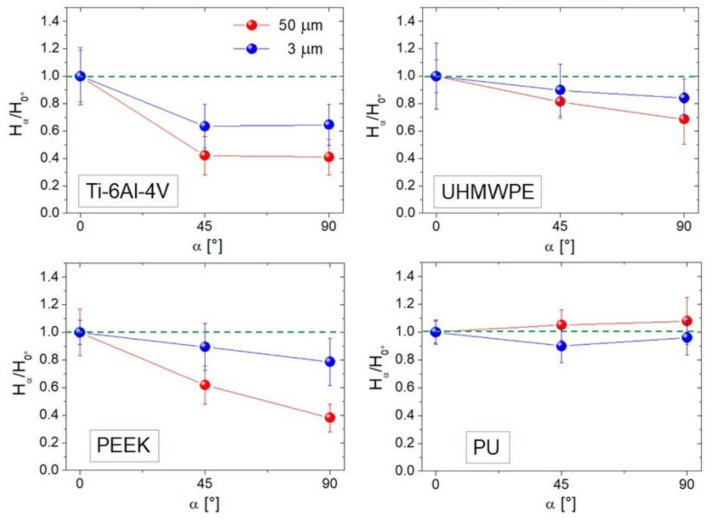
*H_α_/H*_0°_ ratios for *α* = 45° and 90° calculated at *L* = 50 (red curve) and 3 µm (blue curve). The green dotted lines indicate unity (full isotropy) for the reader’s convenience.

We show averaged ratios *H_45°_/H_0°_* and *H_90°_/H_0°_* calculated at two representative image sizes (*L* = 50 and 3 µm) for each sample (Figure 7). *H_α_* was estimated by linearly fitting the high-*k* region of *PSD* functions such as those in Figure 2c; this in practice implies a stronger contribution from distances much smaller than the image size *L*, and reflects the degree of anisotropy expressed by the curves in Figure 7. Of course, Ti-6Al-4V (Figure 7) appears strongly anisotropic for both *L* = 50 and 3 µm, with *H_90°_/H_0°_* ~ 40%–60%; this is acceptable since from Figure 5 one sees that for this surface *S_tr_* is below 0.2 at all distances smaller than *L*. Note, *en passant*, that *H_45°_* ~ *H_90°_* according to what expected with strongly anisotropic surfaces where *H_α_* was predicted to be the same in all angular directions except along the lay (0°), where it will increase [1]. All the other curves follow quite well the mitigation of anisotropy towards small scales seen in Figure 5, that for UHMWPE and PU allows the *H_α_/H_0°_* ratio to approach unity. 

## 4. Discussion

### 4.1. Comparing the Calculated Parameters of a Surface 

In our datasets, spatial anisotropy possesses a slight tendency to mitigate towards small length scales in all samples investigated. This mitigation is reasonable because the scale reduction gives way to the smaller features of contributing to the distribution of asperities within the image, so that depending on the spatial distribution of such features, the evolution of anisotropy will be determined accordingly. These findings confirm the very recent observation of Bartkowiak et al. on the presence of different anisotropies in correspondence with different length scales [4]. In this respect, specific indication can be grasped from the comparison of *S_tr_* (or *H_α_*) and *H_iso_* values, given that these quantities have been evaluated at comparable scale intervals. For example, from the AFM images one notes how face-turning has produced different microstructures on Ti-6Al-4V compared to UHMWPE. Following Table 1, the comparison between the corresponding values of *H_iso_* suggests that the lesser directional morphology of UHMWPE could be associated with its more irregular spatial profile, recognizable from both its lower *H_iso_* (higher ruggedness) and higher error bars of *S_tr_*. An obvious explanation for this is the better machinability of the metal compared to the polymer, resulting in the dominance of the machining upon the morphological features that makes the Ti-6Al-4V’s surface smoother and more regularly patterned compared to UHMWPE. 

On the other hand, the resemblance between the images of Ti-6Al-4V and PEEK suggests that different processing may produce similar morphologies on surfaces different in nature [4]. However, a look at Table 1 points out that despite this apparent resemblance, the semi-finishing of PEEK yielded an overall smoother surface than face-turning did on Ti-6Al-4V. This information cannot be derived simply by comparing the respective topographies or *R_s_* values. At small scales, PEEK exhibits essentially interstitial spherulites, whose random spatial distribution is expected to contribute significantly to decrease the directionality of the surface; this obviously reflects on the *ACF*, which will be affected by such random contribution at least at distances smaller than the average spacing between the lays. However, the height of the features also matters, as it enters into the functions *z*_1_ and *z_2_* of Equation (2). Thus, due to the smoothness of PEEK, its *S_tr_* will increase at slightly shorter distances than PU does, as observed in Figure 5.

### 4.2. Anisotropy Evolution vs. Length Scale

**Figure 8:**
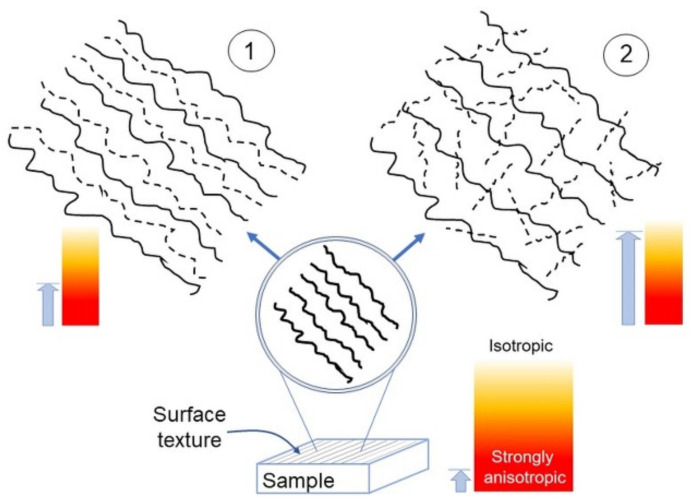
Sketch of the anisotropy appearance in a surface possessing a single lay (bottom), upon magnification in the case 1 (top left) and 2 (top right). The light blue arrows roughly indicate the corresponding anisotropy strength.

Based on the results obtained here, the evolution towards small scales of a given textured surface—this latter schematically represented at the bottom of Figure 8—is likely to follow two ways:

1. Become a nearly deterministic surface because of the strong initial imprint given by the machining (left side of Figure 8). These surfaces may exhibit topographies with spatial periodicity, as Ti-6Al-4V did, even though their most important trait is that their anisotropy tends to persist as the scale decreases. On the other hand, intrinsic characteristics may contribute to mitigate their strong anisotropic character, as seen in the difference between Ti-6Al-4V and UHMWPE.
2. Become a nearly isotropic surface, where the random spatial distribution of asperities dominates the directionality induced by the machining/finishing; this dominance, naturally, contributes to increase *S_tr_* towards small scales. A graphical representation of this fact, otherwise quite difficult to convey, may be given by the “game of pick-up sticks”, where sticks are dropped as a loose bunch onto a tabletop, jumbled into a random pile (right side of Figure 8). These surfaces exhibit a crossover from strongly anisotropic to nearly isotropic behavior. Such a crossover is located at several micrometer-large distances for the samples considered in this work. This latter information may be important in achieving the proper functionality of manufactured surfaces in relation to dimensions relevant to applications; indeed, macroscopically patterned surfaces may also tend to isotropy at the scale of cells or bacteria or, more generally, showing non-negligible fluctuations of anisotropy with scale. As a consequence, it is important that the control over topographical features could be exerted at various scales by means of appropriate machine tools, methods of patterning or chemical and/or physical processes of surface modification. In all these situations, *S_tr_* or *H_α_* may be calculated at relevant length scales, while *H_iso_* can be fruitfully implemented to compare consistently different surfaces, as carried out in this work.

### 4.3. Practical Considerations on H_iso_, S_tr_ and H_α_

We now briefly discuss some practical aspects inherent to the methods used here. As regards the determination of *H_iso_*, we underline that several algorithms other than the “roughness method”—used in this study for its superior numerical accuracy [43]—are available for extracting it from topographic images. For example, box-counting or triangulation algorithms could be used reliably [43,47]; in this case, *H_iso_* will be calculated as an average over several images all with the same (and opportune) size *L*. 

The choice of whether to adopt *S_tr_* or *H_α_* is left to the reader, taking into account the experimental context and the availability of ad hoc algorithms implemented in the software used. Note that, due to its nature of hybrid spatial-height parameter [16], *H_α_* values extracted from single images cannot be compared with *H_iso_* values because software cannot distinguish local regions where, due to finite size of the images and convolution effects, one finds *H* > 1 [36,45,46]. This is most likely the origin of our findings of some values exceeding unity in Figure 7. On the other hand, the relative variations of *H_α_* are likely to be implemented into analysis of spatial anisotropy, as shown by our results; these latter could be refined by calculating *H_α_* through more sophisticated fitting procedures and algorithms, as described in Ref. [45]. 

Finally, it should be underlined that an effective limitation of using *ACF* or *PSD* functions to quantify spatial anisotropy is given, respectively, by the arbitrariness in both thresholding the *ACF* to determine *S_tr_* and when one measures the correct slope *C_α_* to determine *H_α_* from a single *PSD*. These issues may require to improve image sampling, especially on surfaces with strong height variations [43,45,46].

### 4.4. Surfaces with Multiple Lays

Future research directions may use the tools developed in this study to explore multiscale anisotropy of implant surfaces which have undergone gradual smoothing/roughening processes, such as in some of the examples exposed before. For example, Wu et al. studied the effect of roughness on wettability and bacterial adhesion on a machined stainless-steel surface that was originally grooved with about 1 µm-deep grooves and gradually polished by electropolishing [15]. The two most relevant findings of their study were that surface wettability was related to the microtopography of the surface, and to its directionality rather than to its chemical composition. Furthermore, bacterial adhesion increased significantly as roughness decreased or, as it can be readily inferred from their results, as spatial anisotropy changed as a consequence of smoothing the surface. The reader may easily grasp that such a case is conceptually different from the simplification reported in Figure 8. Indeed, the four examples proposed here focused on situations where a single lay or unique direction of anisotropy can be identified, while—typically—the smoothing caused by the superposition of successive processes of finishing/polishing produces additional topographic features with strong directional character that overlap with the original lay. Although common sense may suggest that this circumstance is typical of overall isotropic behavior, as generally accepted [1,2], the results obtained here suggest that the final aspect of the surface should not be taken for granted. Moreover, as a complication of the simple picture of Figure 8, surfaces may possess, rather than a random distribution of features, an intrinsic self-organization or directionality of fibers or clusters, hence contributing to increase spatial anisotropy on a certain scale range. To cover this subject, additional work is required and surfaces possessing such multi-directional lays will deserve attention in the future.

## 5. Conclusions

Our study, carried out on four implant materials with different surface textures, suggests that the strong anisotropic character of their topographies can be well described by a multiscale approach based on solely two parameters, accounting, respectively, for the spatial and vertical variations of the spatial lengths. Within the experimental context investigated in the present work, we showed that this method allows mutual comparison of surfaces with superior accuracy with respect to considering only roughness parameters and is a route to describe quantitatively the complexity of biomaterials’ surfaces after machining or other directional processing. Based on our results, we suggest that a cross-over regime from anisotropy to isotropy may occur, towards small scales, on those surfaces where the inheritance of the machining process is no longer able to prevail over the random spatial distribution of the smaller morphological features. This information may be important in experiments involving the study of the relationship between spatial anisotropy and functional properties of the implants’ topographies in relation to the biological environment. 

## Figures and Tables

**Table 1:** *H_iso_* values obtained from linear fittings of the curves of Figure 3 and the corresponding reduced chi-square (${\tilde{\chi }}^{2})$.

Material	*H_iso_*	${\tilde{\chi }}^{2}$
PEEK	0.891 ± 0.075	4.49 × 10^−3^
Ti-6Al-4V	0.790 ± 0.055	2.39 × 10^−3^
PU	0.733 ± 0.032	0.823 × 10^−3^
UHMWPE	0.661 ± 0.040	1.26 × 10^−3^

## Data Availability

Please refer to suggested Data Availability Statements in section “MDPI Research Data Policies” at https://www.mdpi.com/ethics.
